# Using Lean Six Sigma to Redesign the Supply Chain to the Operating Room Department of a Private Hospital to Reduce Associated Costs and Release Nursing Time to Care

**DOI:** 10.3390/ijerph182111011

**Published:** 2021-10-20

**Authors:** Lisa O’Mahony, Kerrie McCarthy, Josephine O’Donoghue, Seán Paul Teeling, Marie Ward, Martin McNamara

**Affiliations:** 1 Beacon Hospital, UCD Beacon Academy, Beacon Court, Bracken Road, Sandyford Business Park, Sandyford, D18 AK68 Dublin, Ireland; kerrie.mccarthy@beaconhospital.ie (K.M.); josephine.odonoghue@beaconhospital.ie (J.O.); 2 UCD Centre for Interdisciplinary Research, Education and Innovation in Health Systems, School of Nursing, Midwifery and Health Systems, University College Dublin, D04 V1W8 Dublin, Ireland; sean.p.teeling@ucd.ie (S.P.T.); martin.mcnamara@ucd.ie (M.M.); 3 Centre for Person-Centred Practice Research, Division of Nursing, School of Health Sciences, Queen Margaret University Drive, Queen Margaret University, Musselburgh EH21 6UU, East Lothian, Scotland, UK; 4 Centre for Innovative Human Systems, School of Psychology, Trinity College, The University of Dublin, Dublin 2, Ireland; marie.ward@tcd.ie

**Keywords:** supply chain management, healthcare, Lean Six Sigma, 5S, standardisation, stock management, nursing time

## Abstract

Continuity of the supply chain is an integral element in the safe and timely delivery of health services. Lean Six Sigma (LSS), a continuous improvement approach, aims to drive efficiencies and standardisation in processes, and while well established in the manufacturing and supply chain industries, also has relevance in healthcare supply chain management. This study outlines the application of LSS tools and techniques within the supply chain of an Operating Room (OR) setting in a private hospital in Dublin, Ireland. A pre-/post-intervention design was employed following the Define, Measure, Analyse, Improve, Control (DMAIC) framework and applying LSS methodology to redesign the current process for stock management both within the OR storage area and within a pilot OR suite, through collaborative, inclusive, and participatory engagement with staff. A set of improvements were implemented to standardise and streamline the stock management in both areas. The main outcomes from the improvements implemented were an overall reduction in the value of stock held within the operating theatre by 17.7%, a reduction in the value of stock going out of date by 91.7%, and a reduction in the time spent by clinical staff preparing stock required for procedures by 45%, all demonstrating the effectiveness of LSS in healthcare supply chain management.

## 1. Introduction

This paper outlines a case study in a private hospital in Ireland. The hospital operates as a full-service acute hospital with over 200 inpatient beds, 8 Operating Rooms (OR), 1400 healthcare professionals, and 300 consultants. The hospital has an established education and training academy with links to a university-accredited Lean Six Sigma (LSS) training programme. The academy is central to the hospital’s ongoing improvement journey, which is working toward a system-wide deployment of LSS led by its LSS-trained staff. The hospital choose LSS for its approach to improvement as it has been shown that in addition to improving efficiency that it has evidenced a positive impact on patient outcomes, and patient and staff experiences of care [1,2].

The term ‘Lean’ has been used to describe the philosophy of the Toyota Production System (TPS) [3,4,5,6] developed in the car manufacturing industry. Syrett and Lammiman [7] claim that Lean can be seen as a ‘coherent philosophy’ that introduces new ways of working or doing things that can be considered ‘leanness’. Lean is based largely on Taiichi Ohno’s [8] insights, where production activities are either classified as value-adding or waste (non-value-adding), with the purpose to increase the proportion of value-adding activities in a process using methods such as pull, flow, standardised work, leveling, and continuous improvements. Value is based on the end customers’ perception giving an outside reference to a process [3]. Dos Reis Leite and Vieira [6] suggest that health services, as with any service, have issues with quality that are a real challenge for managers and staff, and which lend to Lean for improvement.

Six Sigma is a data-driven process improvement methodology designed to improve process capability and enhance process throughput through the introduction of improvement projects [9,10,11]. It is a quality improvement methodology and management system, focusing on data and costs [12]. Six Sigma is a systematic, data-driven approach using the Define, Measure, Analyse, Improve, and Control (DMAIC) process and utilising design for the Six Sigma method [13]. In the DMAIC model, stakeholder or ‘customer’ engagement is sought from the outset at the Define stage. This stage aims to create value for the customer by identifying problems or issues that need solutions early on [14], utilising the extensive knowledge base of customers and other stakeholders [15]. Both Lean and Six Sigma have a strong focus on the customer, the employee, management support, and teamwork [16].

A hybrid of Lean and Six Sigma as LSS appears in the healthcare literature from 2010 onwards [17] following Lean and Six Sigma integration for project delivery from early 2002 and increased use by 2008. One of the key strengths of LSS is that it seeks to find the ‘root cause’ of problems in a process, which means that it utilises real-time observational data collection [18,19], the process of which is referred to as ‘Gemba’ in Lean terminology [20]. Langabeer and colleagues [21] see Lean as promoting a ‘doing the right thing’ approach (value-added) while Six Sigma focuses on ‘doing things right’ (no errors). LSS focuses on the value desired by the end customer, maintaining continuous flow, continuous improvement, and the elimination of waste/non-value-added activity (e.g., waiting/idle time, excess motion, excess or useless inventory, over-processing, and overproduction [8]. Emerging literature on the benefits of LSS in healthcare can be seen across the health system including but not limited to, improvements in patient length of stay and waiting times [22,23,24], earlier access to diagnostics and treatment [25,26,27], and with an associated reduction in costs and waste [28].

The focus of this paper is on an improvement project within the Operating Room (OR). The OR is where all the elective and emergency surgical activity takes place for specialties including cardiology, neurology, gynaecology, urology, orthopaedics, and gastroenterology. The OR is one of the most resource-intensive areas of a hospital accounting for 52% of its overall expenditure (consumables include a broad range of medical surgical supplies from gauze, syringes, and scissors to sterile customised procedure packs and implantable medical devices). OR consumables rapidly expanded from the hospital opening in 2006 to 2018 in response to new procedures, changes in surgeons, and a more complex case mix. The sharp rise in consumable demand within both the existing defined space and processes led to sporadic storage of stock and inconsistent reordering classification. The Periodic Automatic Replacement (PAR) level is defined as setting a certain quantity and maintaining that level. PAR levels had not been amended in the hospital since 2015. A 2017 December stocktake by the procurement team revealed €27,000 worth of out-of-date stock within the OR which prompted a more detailed examination of the process for stock management and was the catalyst for this improvement project. The hospital had limited insight into out-of-date or wasted stock within the OR clinical working ‘clean’ areas, that are physically demarcated by red lines on the floors, to alert non-OR staff against access and prevent potential cross-contamination. This lack of stock visibility in the existing process for stock management could be attributed to OR procurement staff managing stock only as far as the main OR store room which was located outside the sterile area, effectively beyond the red line. Once the stock was moved over the OR redline to the clinical area, it was no longer monitored. The OR redline was highlighted as a barrier of entry for the procurement team/a move from clean or non-sterile to sterile, with staff required to change clothes and don surgical scrubs and designated footwear to cross the redline.

Overstocking has been shown to significantly drive up annual costs in healthcare systems, with one LSS case study demonstrating excess stock levels in 69 Emergency Room (ER) locations with an associated value of $1,040,000 [29]. The case study identified expired products as a key factor to address in inventory management, estimating that savings of approximately $213,000 yearly could be achieved. It was clear from the literature that over-processing of stock and stock hoarding is a common occurrence in healthcare settings, the fear of supply shortage can create a ‘hoard’ mentality amongst both clinical and supply chain staff, which is in effect a compensatory behaviour for running out of stock [30]. However, it is important to understand that from the clinical staff perspective that out-of-stock situations are undesirable, and not having the correct supplies at Point of Use (POU) when needed can impact on the quality of care [30] and in a worst-case scenario can lead to a loss of life [31], which leads operating theatre staff to err on the side of overstocking. Ensuring stock levels are right is therefore not just an issue of cost or process efficiency, but as indicated, of patient safety. Within the study site, an additional factor was that collating stock for use in surgical cases took an OR nurse on average 4.52 min per case, with the OR nurse pulling up to 11 cases per surgical list, effectively 53 min per day. Given that many OR had 2 lists per day, this time could be doubled. This posed the question ‘Can the use of LSS reduce incidences of overstock, reduce associated costs and release nursing time to care?’

## 2. Materials and Methods

One OR suite which was newly refurbished was chosen as a pilot site to test the use of LSS in reducing overstock, associated costs, and releasing nursing time. The findings of this pilot were subsequently used and translated across all of the other seven ORs in the Hospital.

We utilised a team-based approach with a pre-and post-intervention design employing LSS methods. The team consisted of a multi-disciplinary group of LSS-trained staff involving procurement, quality, and allied therapy professionals.

Aims of this study included

- Standardisation of stock handling over the sterile area redline,
- A reduction in the value of stock going out of date by a minimum of 50%,
- Providing POU stock to avoid out-of-stock situations,
- Creation of dedicated storage areas by surgical and anaesthesia specialty in the main OR stores,
- Remove non-value-added (NVA) activity for nursing staff and release time to care,
- Development of a proof of concept in a pilot OR suite to roll out to the other OR suites, and
- Demonstrate the effectiveness of LSS, with its proven ability to transcend healthcare silos [1,2,19], in improving OR stock issues as part of a whole-system approach to improvement.

We used the Define, Measure, Analyse, Improve, Control (DMAIC) framework to understand and approach the issue of stock and financial waste, raised by the end of year stocktake. Within the framework, we made use of the following LSS tools (Table 1):

We now elaborate on each stage of the improvement using the DMAIC framework to outline our methods.

### 2.1. Define

LSS speaks to ascertaining the Voice of the Customer (VOC) in any process improvement. The VOC in healthcare is considered that of any service user (patient) or provider (staff) or any end-user [1,2], and specifically addresses customer expectations [35]. Obtaining the VOC has also been shown to be synergistic with person-centred approaches to understanding the needs of the person [1,2]. We sought the VOC to firstly identify customers, gather customer needs and then determine issues critical to quality. Stakeholders were identified (Materials manager, OR procurement staff, Chief Financial Officer, OR director of nursing, nurse manager, nurse coordinator, and OR staff nurses). A Responsible, Accountable, Consulted Informed (RACI) tool was used to identify those responsible, those accountable, those who needed to be consulted, and those who need to be kept informed at the different stages of the DMAIC process [38]. For our qualitative semi-structured interviews, we drew on a purposive sample from two main groups working with the stock process—clinical staff in the OR (OR nurse managers, co-coordinators, and staff nurses) and procurement stores staff. The purposive sample was designed to enable data generation and draw inferences and credible explanations from the data that were generated and to be as efficient as practical [39].

The interviews took two forms:

1. A duration of 30 min working alongside individual OR nurses [n = 12] and procurement staff [n = 2] opportunistically (interviewing OR nurses as they dealt with supply and stock for surgical cases).

These interviews took place in the OR, and staff often physically demonstrated issues within the stock room such as the same product in multiple locations or unused stock left in bags or trollies throughout the OR. The open-ended format in which these stakeholders were asked about issues with stock in the OR yielded many comments and these were transcribed and thematically analysed (waste, health, and safety, space, and layout). For example, “thousands worth of aortic cannulas were dumped yesterday and we’re ordering more” (waste) and “we have trollies of stuff everywhere” (space). Concerns were raised about stock remaining exposed in a sterile OR room, and potential blocking of exit doors due to the volume of stock trollies (Health and Safety). When we interviewed the procurement stores staff they reported accountability and ownership issues “we’re relying on busy nurses to return stock to the right place, trollies are wheeled in and left full of stock”. They also reported that once the stock goes over the sterile OR ‘redline’, they do not know what happens to that stock.

2. 40 min meetings with OR managers [n = 8], co-ordinators [n = 4] and OR procurement staff [n = 2]. These interviews were semi-structured and facilitated an interview format that allowed pre-determined topics to be covered (waste, health, and safety, space, and layout) that had been found in the 30 min interviews; however, it also afforded the flexibility to discuss individual participant’s experiences in more detail [40].

Data analysis was carried out using thematic analysis of interview outputs, a common analysis technique for qualitative research [41,42].

A further issue that emerged during VOC 40 min meetings was the over-stocking of sutures. Sutures were in multiple sites and locations within stores and throughout trollies. Stocktake in September 2018 found 1112 boxes of sutures. Having engaged with the supplier, who estimated adequate suture stock for the hospital’s OR needs at 260 boxes, the cost of this over-stock was calculated to be €61,000. Therefore, sutures, originally not considered as part of our improvement, came into the scope of the project following VOC.

**Figure 1:**
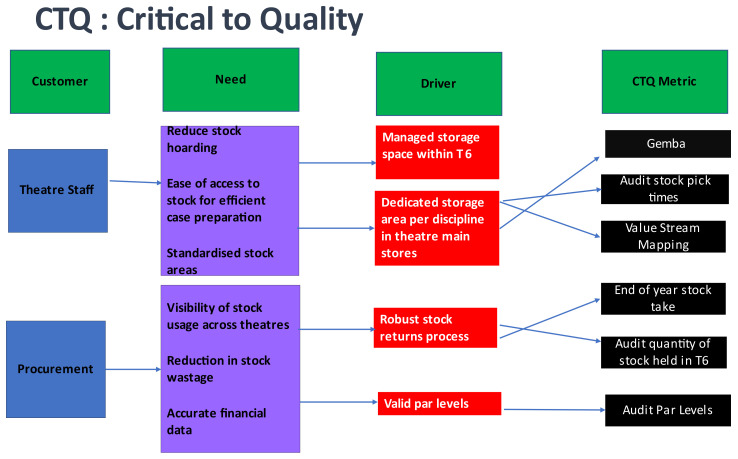
Critical to Quality Tree (CTQ).

Carrying out the VOC enabled us to define factors that were Critical to Quality (CTQ). The CTQ is a tool that structures information collected from customers and translates it into critical and specific process requirements that are measurable [34]. See Figure 1.

The VOC and CTQ exercise identified that the key metrics to focus on for data collection were related to:

1. Nursing time to prepare stock for cases,
2. End of year stocktake, and
3. Value of location of stock within the pilot OR (OR 6).

### 2.2. Measure

To support our data collection, we carried out Gemba walks to fully understand the processes involved from the perspectives of both the nursing staff OR Procurement staff.

**Figure 2:**
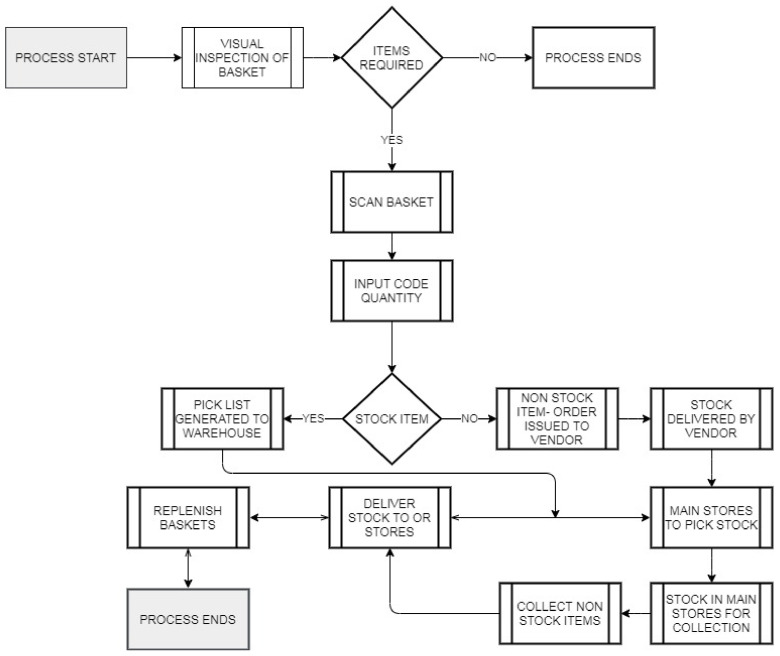
Procurement OR Staff Process Flow Map for Stock Replenishment.

A Gemba walk is a technique used to observe and understand how work is being performed with the following elements: observation (watching people perform the work in-person), location (observing people at the actual location where work is performed), and teaming (interacting with people performing the work) [36]. To ascertain what the current process was we carried out Gemba walks with the OR procurement team (consisting of two staff members) to understand how they managed stock. This enabled us to develop a process flow map of how the OR stock was supplied, stored, collected, prepared, and monitored. Process flow mapping is the graphical representation with illustrative descriptions of how things get done. It facilitates the visualisation of the details of the process and guides decision-making. The process map can identify the major areas of strengths and weaknesses in the existing process, such that the contribution of individual steps in the process. Further, it helps to reduce the cycle times and defects in the process and enhances its productivity [43]. Our current state process map following Gemba illustrated that the OR procurement staff replenished stock twice a day at seven (7) and two (2). See Figure 2.

**Figure 3:**
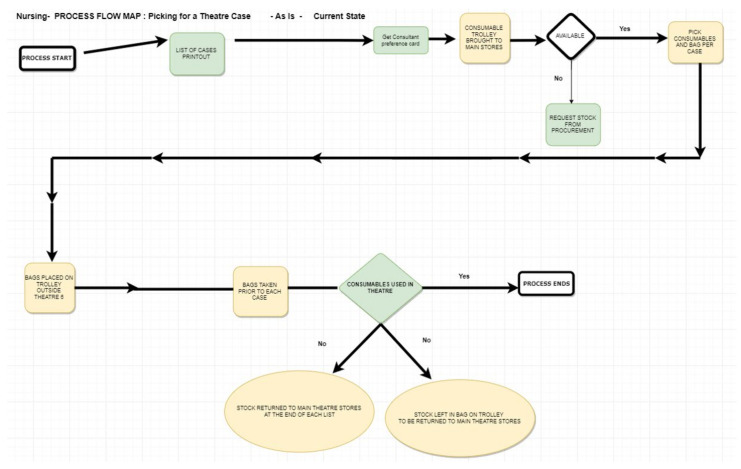
OR Nursing Staff Process for Picking and Returning Stock.

OR nurses gather and collate sterile supplies and materials for every surgical case for each individual consultant surgeon’s operative list. This process is known within the OR as ‘pulling for cases’. Additional Gemba walks were undertaken to observe the process for OR nurses pulling for cases in the main OR storeroom. OR nurses pulled the stock required for each case (listed by individual consultant surgeon preference card) the day before surgery. Over four days, we asked the nurses to document start and end times when pulling stock for cases. See Figure 3.

### 2.3. Analyse

**Figure 4:**
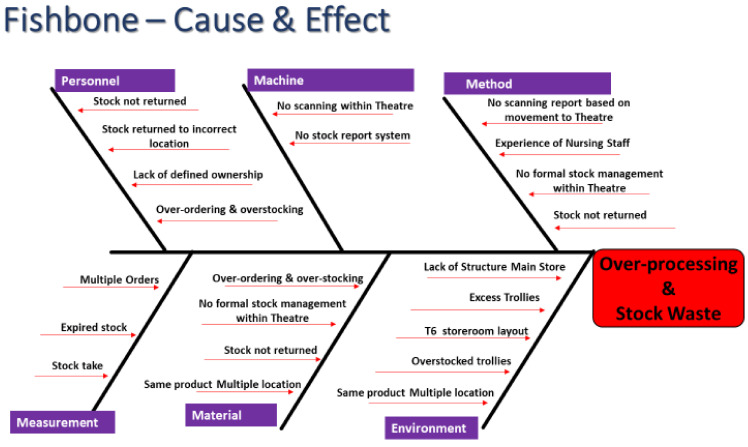
Fishbone for OR Stock Management.

In reviewing the OR procurement staff process map we identified the process of the OR procurement staff traveling to the main stores (Figure 2), searching for stock, and collecting it on a trolley. We identified two return journeys per day and 27 min per journey, a total of 56 min per day. We used an Ishikawa fishbone diagram (Figure 4) to view cause and effect visually. Root causes for over-processing and stock waste included environmental issues (lack of structure in the Main store, overstocked trollies, the same product in multiple locations), lack of formal stock management over the redline, and lack of defined ownership. There was no scanning report for stock once it moved over the redline.

### 2.4. Improve

1. In the improvement phase, we engaged with the key stakeholders from the OR to develop an agreed list and quantity of stock to be held in the pilot OR—number 6. These stakeholders included the clinical nurse manager, deputy clinical nurse manager, and staff nurses (n = 5) from OR 6 (the pilot site). It also included the OR department manager and the OR procurement team (n = 2). OR 6 was chosen as it had recently been refurbished and the improvement in stock management was seen by the OR staff as a way of looking at maximising the new layout of the OR suite. During this engagement, we highlighted the benefits of standardisation of the stock holding in terms of ease of locating stock, having a designated storage space for each item, and agreed minimum reorder points for each item so that supply shortages could be avoided. A stockholding list of products and quantities was agreed and the supply chain management stripped out all the stock, put in place new shelving to create dedicated space for each of the agreed products, and restocked to the agreed quantities.

2. To complement solution 1, Lean 5S was used to organise stock. A key component of our improvement was to organise inventory and facilitate the identification of OR supplies through visual management, specifically colour coding and labeling. The Lean process of 5S is a system to reduce waste and optimise productivity through maintaining an orderly workplace and using visual cues. It is a cyclical methodology of ‘Sort, Set in Order, Shine, Standardise and Sustain the Cycle’ [37]. Lean 5S made it easier and more efficient for staff to identify the products using colour coding and labeling. The stock was organised by discipline, for example, White = Orthopaedics, Blue = Urology and Gynaecology, Green = General Surgery, Yellow = Anaesthetics, and Red = Cardiac. The labeling font on the baskets was increased so that it was more easily identifiable.

3. The hospital procurement manager agreed to remove the collection process of stock from the main store from the OR procurement team that we had identified in the process map. They felt that the return on investment on this change of process would be that the 27 min identified per day per OR procurement team member (n = 2) could be used to better effect managing the stock across the OR redline and obviate out-of-date stock occurrences. The OR procurement team introduced ‘returns baskets’ to manage unused stock returns post-surgical case lists.

4. Our review of stock included a review of suture supplies. We reviewed the ‘as-is’ or current state process of suture storage and found sutures were stored in multiple locations within each of the operating theatres and also held on several different stock trollies across the OR redline. Engaging with the key stakeholders in OR it was agreed to establish one centralised suture storage area in the theatre main corridor. During the engagement, we referred back to our CTQ and VOC to highlight the benefits of centralised storage in addressing their needs to reduce stock hoarding and enable ease of access to stock for case preparation. It was agreed that we would work with the supplier representative to review the need for such a wide variety of suture codes to streamline the codes and reduce stock holding. Following the current state review the supplier representative estimated for our number of theatres, cases, and case-mix we should be holding approximately 408 boxes and 180 codes. The supplier representative engaged with the consultants to discuss their current suture preferences explained the similarity between codes and got consensus from consultants to move to a condensed list of codes.

5. The Red Line demarcating the sterile of clean areas was, with the agreement of the Infection Prevention and Control Team, moved to allow the main OR storeroom to become part of the sterile environment. This, with their agreement and endorsement, effectively moved the OR Procurement team into the sterile area and surgical scrubs. This was seen as being inclusive and person-centred and synergistic with the Lean philosophy of respect for person [1,2].

Identified and implemented solutions are summarised in Table 2.

## 3. Results

Having put in place the process improvements outlined above, we reanalysed the data examined in our Define and Measure phases to determine the real impact of the changes put in place and how they addressed the aims in our project charter.

### 3.1. Reduction in Stock Holding Value

One of the key issues highlighted in our data analysis was the lack of insight into stock handling over the OR red line. As discussed in our solutions above we analysed the stock holding in one of the eight OR suites and developed a proof of concept for roll out across the other seven OR suites. A full stocktake was carried out in the pilot OR and there was a total value of €221,052 worth of stock in that OR store, with the same product found in multiple locations (see Table 3).

When the annual stocktake was repeated in December 2018 the stock holding was valued at €181,913, a 17.7% reduction.

Pre-intervention suture stock was stored in multiple locations within each OR. A full stocktake of all sutures across the eight OR suites was conducted to establish the quantity and range of product codes, a total of 1112 boxes and 247 codes was recorded (see Table 4).

Following the creation of centralised suture storage, there was an initial 33% reduction in stock.

A further stocktake was carried out six months post-intervention to analyse the impact of the improvement and stock holding had reduced again to 53% reduction from pre-improvement.

### 3.2. Reduction in OR Nursing Stock Prep Time

**Figure 5:**
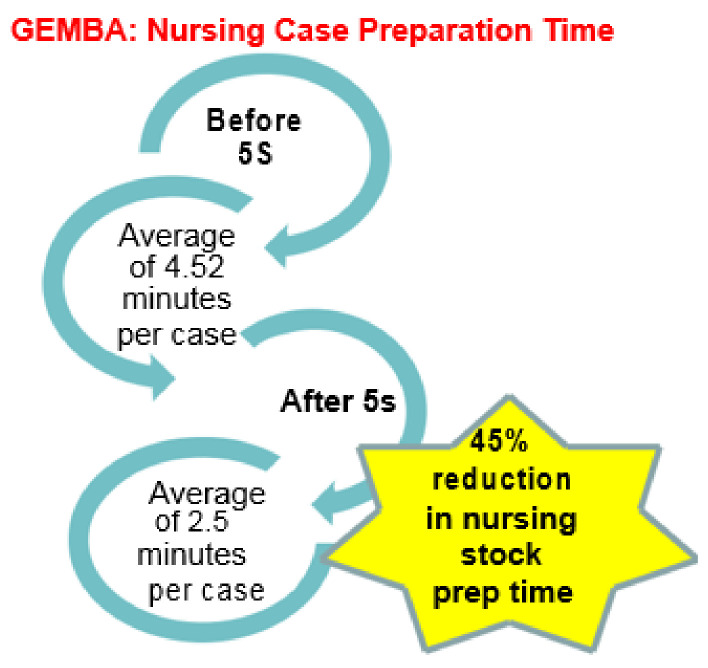
Virtual Gemba Data.

Pre-improvement, the pulling of consumable stock for cases took on average 4.52 min per case. The OR nurse could pull stock for up to 11 cases per surgical procedure list. Post-5S, where a colour-coded dedicated storage area for each discipline was created, we asked the nursing staff to repeat this exercise (see Figure 5). The average stock preparation time per case was reduced to 2.5 min per case, a 45% reduction in time utilised for this non-clinical task. Based on post-intervention VOC with OR nursing staff we established that this released time did not directly translate into the earlier finish of OR operative lists, due to the variation, complexity, and specific requirements in surgical specialties. However, they expressed that the released time was valuable to nurses in caring for peri-operative patients within the department.

### 3.3. Reduction in Value of Stock Going out of Date

Our initial goal was the reduction of stock going out of date by at least 50%. During the annual end-of-year stocktake in December 2017, it was revealed there was €27,000 worth of out-of-date stock located in the OR main storeroom; see Table 5. Post-5S, there was a dedicated space for stock items, with clearer labeling and colour coding for ease of identifying products. The stocktake in December 2018 revealed €2231 worth of out-of-date stock, a reduction of 91.7%; see Table 5.

## 4. Discussion

We established that over-processing of stock and stock hoarding occurs commonly in healthcare [30]. However, we also identified that having stock at POU is essential to ensure the quality of care [30,31]. There is therefore a cost to healthcare organisations both in monetary terms of over-purchasing stock/stock going out of date and also the human cost of over-processing of orders and in the case of our study nursing time spent on non-clinical tasks. This study was successful in both reducing stock going out of date, releasing time to care, and used Lean 5S, shown to be effective in clinical settings [44], to facilitate visual management and place stock at POU.

Early engagement with key stakeholders, particularly those on the ground carrying out the stock management tasks (both clinical and non-clinical) is critical to getting buy-in to any change. Following the DMAIC process enabled the team to present data-driven areas for potential improvements and highlight the impact these improvements made in their day-to-day tasks. We found that stakeholder engagement required a deep appreciation of the system [45], in which inquiries are conducted and improvements implemented, which was critical to the effective use of the LSS improvement approach in this project. These approaches enabled us to build team relationships among ourselves as an improvement team, enabled collaborative working with our stakeholders towards a shared vision and future, and provided a coherent and structured framework to identify and address the problems faced by the team. LSS tools provided an invaluable way to clarify and detail the problem at hand, and to pinpoint where solutions should be focused. Throughout the process, they served to keep the focus on what would add the greatest value to the team and the service. To avoid reducing our engagement with our stakeholders to a checklist exercise, we continuously drew on person-centred collaborative, inclusive, and participatory (CIP) principles [46] to inform our thinking about how we could work with colleagues to evaluate and improve our service.

The feedback gathered in the VOC pre-and post-implementation showed that while the new stock layout post-5S would “take getting used to”, staff could clearly see the benefits of a streamlined approach to storage. A key learning from our study is that early and frequent engagement with the stakeholders carrying out the day-to-day tasks was critical to success and highlights the benefit qualitative data-gathering exercises such as VOC can bring. Much of the literature we reviewed on stock management in clinical settings relied heavily on the quantitative data only [29]. Having said that, the quantitative data were very beneficial in highlighting to senior management the financial and time-saving benefits of the application of LSS in our OR department.

Having successfully implemented the proof of concept for stock management within OR suite 6, this has now been rolled out to the other seven ORs. This upscaling from one OR to all eight ORs was only possible by removing the task of the procurement staff traveling to the warehouse to pick and collect the stock, a time saving of 54 min per staff member. Being able to show the time saving of removing this task from procurement staff to utilise in the management of stock across the eight theatres created buy into the mindset of a change in practice rather than additional workload. A further success factor from this project is that it served as a platform for future improvement, with a 2nd generation project undertaken to further release nursing time resulting in a 55% decrease in overall nursing time spent in gathering and preparing materials for surgical cases. The overall OR nurse time saved in the process for surgical preparation per case was 16 min 45 s or 55%. Building on our own work, the number of storage locations (touchpoints) the OR nurse had to access to collect materials was reduced by 66% from a total of 98 touchpoints to 38 touchpoints. This 2nd generation project is currently in publication. The results of both studies indicate that LSS has provided a platform for continuous improvement and contributes to the literature on the use of LSS for process and quality improvement in hospitals [47]. This study is not without limitations. While the process improvement was evaluated and piloted within only one OR, the research findings offer opportunities for reflection, learning, and development for teams working within busy OR departments. Additionally, since the completion of this project, the pilot has had an incremental role out to the 7 other OR suites, and as discussed, there has been second generation LSS work within the OR on further improving stock management and releasing nursing time to care. We also acknowledge that this process improvement, although impacting on patients through releasing nursing time to care and access to stock, did not engage with surgical patients.

## 5. Conclusions

The results realised from our study demonstrate the benefits LSS principles can bring to stock management within the OR and indeed wider hospital settings. In particular, the application of the 5S approach to storage within our OR main stores and pilot OR highlights the efficiencies that can be achieved in terms of time savings for clinical staff and reduction in stock wastage. It also illustrates that although stock management may seem divorced from the role of the OR nurse, it has implications for nurses in the time they have to spend with their patients and on their nursing role. It demonstrates that LSS methodologies can be seen as more than what McNamara and Teeling [48] refer to as a decontextualised toolkit, and emphasises its use for engaging with all people involved in the complex processes inherent in healthcare across the entire health system.

## Figures and Tables

**Table 1:** LSS tools used by the team.

Improvement Tool	Description	Reference Number
Project Charter	A project charter defines the problem statement and attains baseline data for the project. Used to identify goals of the project and what is in scope	[32]
SMART Goals Specific, Measurable, Achievable, Relevant, and Timebound	SMART is used to manage the project goals, to determine if they are Specific, Measurable, Achievable, Relevant and Timebound	[32]
SIPOC Suppliers, Inputs, Process, Outputs, and Customers	A high-level SIPOC (Supplier, Input, Process, Output, and Customer) highlights the process steps and defines the customers and stakeholders	[33]
RACI Responsible, Accountable, Consulted Informed	Identifies which stakeholders are responsible, accountable, which need to be kept informed or consulted	[32]
CTQ Critical To Quality	The CTQ is designed to capture the key measurable characteristics of a process or service whose performance standards must be met to satisfy the service user	[34]
VOC Voice of the Customer	Engaging with the customer to gather their feedback about their experiences with and expectations for your products or services	[1,2,35]
Gemba	Observation of the actual process taking place	[36]
Fishbone	Identifies root causes, representing the effect and the factors or causes influencing it	[32]
5S	A system to reduce waste and optimise productivity through maintaining an orderly workplace and using visual cues. A cyclical methodology of ‘Sort, Set in Order, Shine, Standardise and Sustain the Cycle’	[37]

**Table 2:** Identified and implemented solutions.

Standardisation for Stock Handling—Solutions	
A 5S process carried out in OR stock room—visual management	Relocation of the OR red-line demarcating sterile areas to facilitate stock management
A 5S process carried out in OR 6—visual management	Introduction of returns baskets to facilitate stock management post-surgical case list
Implementation of centralised suture storage	Inclusion of the OR procurement team as part of the overall OR team within the department
A condensed list of suture codes	

**Table 3:** Stocktake value in pilot OR pre-and post-intervention.

Description	Value
Stock value pre-intervention October 2018	€221,052
Stock value post-intervention December 2018	€181,913
Percentage reduction %—post-intervention	17.7%

**Table 4:** Suture stock value.

	Boxes	Codes	Percentage Reduction %
Stock Count Pre-Intervention September 2018	1112 Boxes	247 Codes	
Stock Count Post-Intervention March 2019	741 Boxes	194 codes	33%
Stock Count 6 Months Post-Intervention September 2019	518 Boxes	187 codes	53%

**Table 5:** Main storeroom end-of-year stocktakes.

Description	Value
Stock value pre-intervention December 2017	€27,000
Stock value post-intervention December 2018	€2231
Percentage reduction %—post-intervention	91.7%

## Data Availability

The data presented in this study are available in the paper.
